# Supplementary material for: Sense of control buffers against stress
Source: eLife. 2026 Feb 10;14:RP105025. doi: 10.7554/eLife.105025 (PMC12890248; doi:10.7554/eLife.105025)
Supplement: Supplementary file 1. [file elife-105025-supp1.docx]

**Supplementary File 1.** ICC results using a 1^st^/2^nd^ half split of the data

To test a different data parcellation for the split half analyses for the ICC calculations, we did a 1^st^ half/2^nd^ half split of the slider measures. These values are slightly lower than the odd/even split in the main text, likely due to small drifts in task performance as participants become more familiar with the task (control and difficulty sliders), especially because Study 2 was designed to experience increased control throughout the WS task due to the task parameters chosen.

***Control sliders***: Study 1: ICC(A,1) = 0.823 [95% CI: 0.792, 0.850], *F*(472,472) = 10.30*,* *p*<.001; Study 2: ICC(A,1) = 0.679 [95% CI: 0.528, 0.777], *F*(200,44.2) = 6.04*,* *p*<.001.

***Difficulty sliders***: Study 1: ICC(A,1) = 0.842 [95% CI: 0.807, 0.870], *F*(472,257) = 12.1*,* *p*<.001; Study 2: ICC(A,1) = 0.567 [95% CI: 0.269, 0.732], *F*(200,16.6) = 4.75*,* *p*<.001.

***Stress sliders***: Study 1: ICC(A,1) = 0.448 [95% CI: 0.373, 0.517], *F*(472,470) = 2.63*,* *p*<.001; Study 2: ICC(A,1) = 0.71 [95% CI: 0.648, 0.762], *F*(294,294) = 5.88.
