# Supplementary material for: Sense of control buffers against stress
Source: eLife. 2026 Feb 10;14:RP105025. doi: 10.7554/eLife.105025 (PMC12890248; doi:10.7554/eLife.105025)
Supplement: Supplementary file 2. — (A) Associations between questionnaire scores and mean task-level subjective control in Study 1, with WS control condition included as a covariate in the linear model. Adjusted p values (padj.) are FDR corrected p values given we ran five different models. (B) Associations between questionnaire scores and mean task-level subjective control in Study 2. The control condition was not included as a covariate in the linear model because the WS task was only presented in High Control. Adjusted p values (padj.) are FDR corrected p values given we ran five different models. (C) Associations between questionnaire scores and estimated intercept parameter from the computational model predicting control from WS task parameters in Study 1, with WS task control condition included as a covariate in the linear model. Adjusted p values (padj.) are FDR corrected p values given we ran five different models. (D) Associations between questionnaire scores and estimated intercept parameter from the computational model predicting control from WS task parameters in Study 2. Adjusted p values (padj.) are FDR corrected p values given we ran five different models. (E) Associations between questionnaire scores and mean task-level stress ratings, with external stressor intensity condition included as a covariate in the linear model for Study 1. Adjusted p values (padj.) are FDR corrected p values given we ran five different models. (F) Associations between questionnaire scores and mean task-level stress ratings, with external stressor intensity condition included as a covariate in the linear model for Study 2. Adjusted p values (padj.) are FDR corrected p values given we ran five models. [file elife-105025-supp2.docx]

**Supplementary File 2A.** Associations between questionnaire scores and mean task-level subjective control in Study 1, with WS control condition included as a covariate in the linear model. Adjusted p values (*p_adj._*) are FDR corrected p values given we ran five different models.

|  | **Study 1 - Mean Subjective Control** | | | | | | | | | | |
| --- | --- | --- | --- | --- | --- | --- | --- | --- | --- | --- | --- |
|  | **Model 1 –**  **STAI State** | | **Model 2 –**  **LOC** | | **Model 3 –**  **STAI Trait** | | **Model 4 –**  **PHQ** | | **Model 5 –**  **SPIN** | | |
| *Predictors* | *Estimates*  *(95% CI)* | *p*  *(p_adj._)* | *Estimates*  *(95% CI)* | *p*  *(p_adj._)* | *Estimates*  *(95% CI)* | *p*  *(p_adj._)* | *Estimates*  *(95% CI)* | *p*  *(p_adj._)* | *Estimates*  *(95% CI)* | *p*  *(p_adj._)* |  |
| (Intercept) | 72.17 (66.82 – 77.53) | **<.001**  **(<.001)** | 65.79 (59.11 – 72.47) | **<.001**  **(<.001)** | 73.52 (67.58 – 79.45) | **<.001**  **(<.001)** | 65.27 (61.80 – 68.75) | **<.001**  **(<.001)** | 63.12 (59.38 – 66.85) | **<.001**  **(<.001)** |  |
| WS Task Control [Low] | -20.69 (-23.53 – -17.85) | **<.001**  **(<.001)** | -19.42 (-22.92 – -15.92) | **<.001**  **(<.001)** | -20.73 (-23.57 – -17.89) | **<.001**  **(<.001)** | -19.83 (-23.30 – -16.35) | **<.001**  **(<.001)** | -19.52 (-23.05 – -16.00) | **<.001**  **(<.001)** |  |
| STAI State (initial) | -0.25 (-0.39 – -0.12) | **<.001**  **(<.001)** |  |  |  |  |  |  |  |  |  |
| LOC |  |  | -0.30 (-0.76 – 0.15) | .191  (.239) |  |  |  |  |  |  |  |
| STAI Trait |  |  |  |  | -0.24 (-0.36 – -0.12) | **<.001**  **(<.001)** |  |  |  |  |  |
| PHQ |  |  |  |  |  |  | -0.50 (-0.84 – -0.16) | **.004**  **(.007)** |  |  |  |
| SPIN |  |  |  |  |  |  |  |  | -0.06 (-0.18 – 0.06) | .350  (.350) |  |
| Observations | 470 | | 298 | | 467 | | 298 | | 296 | | |
| R^2^ / R^2^ adjusted | 0.322 / 0.319 | | 0.290 / 0.286 | | 0.325 / 0.322 | | 0.306 / 0.301 | | 0.292 / 0.287 | | |

**Supplementary File 2B.** Associations between questionnaire scores and mean task-level subjective control in Study 2. Control condition was not included as a covariate in the linear model because the WS task was only presented in High Control. Adjusted p values (*p_adj._*) are FDR corrected p values given we ran five different models.

|  | **Study 2 - Mean Subjective Control** | | | | | | | | | |
| --- | --- | --- | --- | --- | --- | --- | --- | --- | --- | --- |
|  | **Model 1 –**  **STAI State** | | **Model 2 –**  **LOC** | | **Model 3 –**  **STAI Trait** | | **Model 4 –**  **PHQ** | | **Model 5 –**  **SPIN** | |
| *Predictors* | *Estimates*  *(95% CI)* | *p*  *(p_adj._)* | *Estimates*  *(95% CI)* | *p*  *(p_adj._)* | *Estimates*  *(95% CI)* | *p*  *(p_adj._)* | *Estimates*  *(95% CI)* | *p*  *(p_adj._)* | *Estimates*  *(95% CI)* | *p*  *(p_adj._)* |
| (Intercept) | 77.21 (69.80 – 84.62) | **<.001**  **(<.001)** | 73.49 (64.74 – 82.23) | **<.001**  **(<.001)** | 78.89 (70.39 – 87.39) | **<.001**  **(<.001)** | 65.74 (62.22 – 69.27) | **<.001**  **(<.001)** | 69.28 (65.13 – 73.42) | **<.001**  **(<.001)** |
| STAI State (initial) | -0.39 (-0.58 – -0.21) | **<.001**  **(<.001)** |  |  |  |  |  |  |  |  |
| LOC |  |  | -0.80 (-1.39 – -0.21) | **.008**  **(.008)** |  |  |  |  |  |  |
| STAI Trait |  |  |  |  | -0.37 (-0.55 – -0.19) | **<.001**  **(<.001)** |  |  |  |  |
| PHQ |  |  |  |  |  |  | -0.55 (-0.94 – -0.16) | **.006**  **(.008)** |  |  |
| SPIN |  |  |  |  |  |  |  |  | -0.34 (-0.50 – -0.17) | **<.001**  **(<.001)** |
| Observations | 199 | | 198 | | 196 | | 201 | | 198 | |
| R^2^ / R^2^ adjusted | 0.084 / 0.079 | | 0.035 / 0.030 | | 0.077 / 0.072 | | 0.037 / 0.032 | | 0.078 / 0.074 | |

**Supplementary File 2C –** Associations between questionnaire scores and estimated intercept parameter from the computational model predicting control from WS task parameters in Study 1, with WS task control condition included as a covariate in the linear model. Adjusted p values (*p_adj._*) are FDR corrected p values given we ran five different models.

| **Study 1 – intercept parameter estimate from computational model** | | | | | | | | | | | | | | | |
| --- | --- | --- | --- | --- | --- | --- | --- | --- | --- | --- | --- | --- | --- | --- | --- |
|  | **Model 1 –**  **STAI State** | | | **Model 2 –**  **LOC** | | **Model 3 –**  **STAI Trait** | | | **Model 4 –**  **PHQ** | | | **Model 5 –**  **SPIN** | | | |
| *Predictors* | *Estimates*  *(95% CI)* | *p*  *(p_adj._)* | *Estimates*  *(95% CI)* | | *p*  *(p_adj._)* | | *Estimates*  *(95% CI)* | *p*  *(p_adj._)* | | *Estimates*  *(95% CI)* | *p*  *(p_adj._)* | | *Estimates*  *(95% CI)* | *p*  *(p_adj._)* |  |
| (Intercept) | 67.72 (62.60 – 72.85) | **<.001**  (**<.001)** | 63.82 (57.35 – 70.29) | | **<.001**  (**<.001)** | | 69.22 (63.55 – 74.89) | **<.001**  (**<.001)** | | 62.79 (59.42 – 66.16) | **<.001**  (**<.001)** | | 60.84 (57.22 – 64.45) | **<.001**  (**<.001)** |  |
| WS Task Control [Low] | -19.88 (-22.60 – -17.16) | **<.001**  **(<.001)** | -18.80 (-22.19 – -15.40) | | **<.001**  **(<.001)** | | -19.88 (-22.59 – -17.16) | **<.001**  **(<.001)** | | -19.17 (-22.54 – -15.79) | **<.001**  **(<.001)** | | -18.89 (-22.31 – -15.48) | **<.001**  **(<.001)** |  |
| STAI State (initial) | -0.21 (-0.34 – -0.09) | **.001**  **(.003)** |  | |  | |  |  | |  |  | |  |  |  |
| LOC |  |  | -0.32 (-0.76 – 0.12) | | .155  (.194) | |  |  | |  |  | |  |  |  |
| STAI Trait |  |  |  | |  | | -0.21 (-0.33 – -0.09) | **<.001**  **(.002)** | |  |  | |  |  |  |
| PHQ |  |  |  | |  | |  |  | | -0.46 (-0.79 – -0.13) | **.007**  **(.011)** | |  |  |  |
| SPIN |  |  |  | |  | |  |  | |  |  | | -0.05 (-0.17 – 0.06) | .364  (.364) |  |
| Observations | 470 | | | 298 | | 467 | | | 298 | | | 296 | | | |
| R^2^ / R^2^ adjusted | 0.320 / 0.317 | | | 0.290 / 0.286 | | 0.324 / 0.321 | | | 0.303 / 0.299 | | | 0.291 / 0.286 | | | |

**Supplementary File 2D –** Associations between questionnaire scores and estimated intercept parameter from the computational model predicting control from WS task parameters in Study 2. Adjusted *p* values (*p_adj._*) are FDR corrected *p* values given we ran five different models.

| **Study 2 – intercept parameter estimate from computational model** | | | | | | | | | | |
| --- | --- | --- | --- | --- | --- | --- | --- | --- | --- | --- |
|  | **Model 1 –**  **STAI State** | | **Model 2 –**  **LOC** | | **Model 3 –**  **STAI Trait** | | **Model 4 –**  **PHQ** | | **Model 5 –**  **SPIN** | |
| *Predictors* | *Estimates*  *(95% CI)* | *p*  *(p_adj._)* | *Estimates*  *(95% CI)* | *p*  *(p_adj._)* | *Estimates*  *(95% CI)* | *p*  *(p_adj._)* | *Estimates*  *(95% CI)* | *p*  *(p_adj._)* | *Estimates*  *(95% CI)* | *p*  *(p_adj._)* |
| (Intercept) | 70.06 (63.18 – 76.95) | **<.001**  **(<.001)** | 68.00 (59.92 – 76.07) | **<.001**  **(<.001)** | 72.52 (64.69 – 80.36) | **<.001**  **(<.001)** | 60.39 (57.14 – 63.64) | **<.001**  **(<.001)** | 63.10 (59.25 – 66.95) | **<.001**  **(<.001)** |
| STAI State (initial) | -0.35 (-0.52 – -0.18) | **<.001**  **(<.001)** |  |  |  |  |  |  |  |  |
| LOC |  |  | -0.79 (-1.33 – -0.25) | **.005**  **(.005)** |  |  |  |  |  |  |
| STAI Trait |  |  |  |  | -0.35 (-0.51 – -0.18) | **<.001**  **(<.001)** |  |  |  |  |
| PHQ |  |  |  |  |  |  | -0.55 (-0.91 – -0.19) | **.003**  **(.004)** |  |  |
| SPIN |  |  |  |  |  |  |  |  | -0.30 (-0.45 – -0.15) | **<.001**  **(<.001)** |
| Observations | 199 | | 198 | | 196 | | 201 | | 198 | |
| R^2^ / R^2^ adjusted | 0.077 / 0.072 | | 0.040 / 0.035 | | 0.080 / 0.075 | | 0.043 / 0.038 | | 0.073 / 0.068 | |

**Supplementary File 2E.** Associations between questionnaire scores and mean task-level stress ratings, with external stressor intensity condition included as a covariate in the linear model for Study 1. Adjusted p values (*p_adj._*) are FDR corrected p values given we ran five different models.

|  | **Study 1 - Mean Subjective Stress** | | | | | | | | | |
| --- | --- | --- | --- | --- | --- | --- | --- | --- | --- | --- |
|  | **Model 1 –**  **STAI State** | | **Model 2 –**  **LOC** | | **Model 3 –**  **STAI Trait** | | **Model 4 –**  **PHQ** | | **Model 5 –**  **SPIN** | |
| *Predictors* | *Estimates (95% CI)* | *p*  *(p_adj._)* | *Estimates (95% CI)* | *p*  *(p_adj._)* | *Estimates*  *(95% CI)* | *p*  *(p_adj._)* | *Estimates (95% CI)* | *p*  *(p_adj._)* | *Estimates (95% CI)* | *p*  *(p_adj._)* |
| (Intercept) | 2.39 (-3.56 – 8.35) | .430  (.538) | 33.70 (25.15 – 42.25) | **<.001**  **(<.001)** | 1.07 (-5.52 – 7.67) | .750  (.750) | 29.77 (25.84 – 33.70) | **<.001**  **(<.001)** | 32.33 (27.88 – 36.78) | **<.001**  **(<.001)** |
| Stressor Intensity [Low] | -3.10 (-6.25 – 0.05) | .054  (.089) | -4.91 (-9.70 – -0.11) | **.045**  (.089) | -4.90 (-8.08 – -1.72) | **.003**  **(.013)** | -1.06 (-5.43 – 3.30) | 0.632  (0.632) | -4.04 (-8.61 – 0.52) | .082  (.103) |
| STAI State (initial) | 1.06 (0.92 – 1.21) | **<.001**  **(<.001)** |  |  |  |  |  |  |  |  |
| LOC |  |  | 0.75 (0.16 – 1.34) | **.013**  **(.013)** |  |  |  |  |  |  |
| STAI Trait |  |  |  |  | 0.92 (0.78 – 1.05) | **<.001**  **(<.001)** |  |  |  |  |
| PHQ |  |  |  |  |  |  | 1.88 (1.47 – 2.28) | **<.001**  **(<.001)** |  |  |
| SPIN |  |  |  |  |  |  |  |  | 0.47 (0.32 – 0.61) | **<.001**  **(<.001)** |
| Observations | 470 | | 298 | | 467 | | 298 | | 296 | |
| R^2^ / R^2^ adjusted | 0.324 / 0.321 | | 0.035 / 0.029 | | 0.297 / 0.294 | | 0.232 / 0.227 | | 0.132 / 0.126 | |

**Supplementary File 2F.** Associations between questionnaire scores and mean task-level stress ratings, with external stressor intensity condition included as a covariate in the linear model for Study 2. Adjusted p values (*p_adj._*) are FDR corrected p values given we ran five models.

|  | **Study 2 - Mean Subjective Stress** | | | | | | | | | |
| --- | --- | --- | --- | --- | --- | --- | --- | --- | --- | --- |
|  | **Model 1 –**  **STAI State** | | **Model 2 –**  **LOC** | | **Model 3 –**  **STAI Trait** | | **Model 4 –**  **PHQ** | | **Model 5 –**  **SPIN** | |
| *Predictors* | *Estimates (95% CI)* | *p*  *(p_adj._)* | *Estimates (95% CI)* | *p*  *(p_adj._)* | *Estimates*  *(95% CI)* | *p*  *(p_adj._)* | *Estimates (95% CI)* | *p*  *(p_adj._)* | *Estimates (95% CI)* | *p*  *(p_adj._)* |
| (Intercept) | -10.00 (-16.61 – -3.40) | **.003**  **(.004)** | 17.59 (7.74 – 27.43) | **<.001**  **(<.001)** | -11.97 (-20.28 – -3.65) | **.005**  **(.005)** | 23.93 (19.81 – 28.05) | **<.001**  **(<.001)** | 23.19 (18.00 – 28.37) | **<.001**  **(<.001)** |
| Stressor Intensity [Low] | -0.80 (-4.50 – 2.90) | .671  (.734) | -2.09 (-7.01 – 2.84) | .404  (.734) | -1.17 (-5.25 – 2.91) | .574  (.734) | -0.73 (-4.97 – 3.51) | .734  (.734) | -1.18 (-5.81 – 3.46) | .618  (.734) |
| STAI State (initial) | 1.27 (1.12 – 1.42) | **<.001**  **(<.001)** |  |  |  |  |  |  |  |  |
| LOC |  |  | 1.58 (0.94 – 2.22) | **<.001**  **(<.001)** |  |  |  |  |  |  |
| STAI Trait |  |  |  |  | 1.12 (0.95 – 1.29) | **<.001**  **(<.001)** |  |  |  |  |
| PHQ |  |  |  |  |  |  | 2.21 (1.83 – 2.59) | **<.001**  **(<.001)** |  |  |
| SPIN |  |  |  |  |  |  |  |  | 0.74 (0.57 – 0.91) | **<.001**  **(<.001)** |
| Observations | 293 | | 292 | | 290 | | 295 | | 290 | |
| R^2^ / R^2^ adjusted | 0.480 / 0.477 | | 0.079 / 0.073 | | 0.377 / 0.372 | | 0.310 / 0.305 | | 0.200 / 0.194 | |
