# Supplementary material for: Sense of control buffers against stress
Source: eLife. 2026 Feb 10;14:RP105025. doi: 10.7554/eLife.105025 (PMC12890248; doi:10.7554/eLife.105025)
Supplement: Supplementary file 3. — (A) Relationship between subjective control, perceived difficulty and subjective stress during the WS Task in Study 2, also when removing the final WS timepoint and including Domain, or when including win rate. Predicted values from the leftmost column (Subjective Stress) model are presented in Figure 2. (B) Excluding the final timepoint to investigate the effects of control, difficulty and stress during the WS Task for Study 1 (left-hand model). Including all timepoints (as original model) and additionally including overall win rate as a covariate for Study 1 (right hand model). [file elife-105025-supp3.docx]

**Supplementary File 3A.** Relationship between subjective control, perceived difficulty and subjective stress during the WS Task in Study 2, also when removing the final WS timepoint and including Domain, or when including win rate. Predicted values from the leftmost column (Subjective Stress) model are presented in Figure 2.

|  | **Subjective Stress** | | **Subjective Stress**  **(excl. final timepoint)** | | | **Subjective Stress**  **(including win rate)** | | **Subjective Stress**  **(including Domain)** | | **Subjective Stress**  **(including Domain, excl. final timepoint)** | |
| --- | --- | --- | --- | --- | --- | --- | --- | --- | --- | --- | --- |
| *Predictors* | *Estimates*  *(95% CI)* | *p* | *Estimates*  *(95% CI)* | | *p* | *Estimates*  *(95% CI)* | *p* | *Estimates*  *(95% CI)* | *p* | *Estimates*  *(95% CI)* | *p* |
| (Intercept) | 45.13 (35.99 – 54.28) | **<.001** | 40.82 (30.29 – 51.35) | | **<.001** | 53.01 (31.51 – 74.50) | **<.001** | 40.04 (30.55 – 49.53) | **<.001** | 36.66 (25.98 – 47.34) | **<.001** |
| Subjective Control | -0.33 (-0.43 – -0.23) | **<.001** | -0.41 (-0.52 – -0.29) | | **<.001** | -0.32 (-0.43 – -0.22) | **<.001** | -0.31 (-0.42 – -0.21) | **<.001** | -0.39 (-0.50 – -0.27) | **<.001** |
| Perceived Difficulty | 0.32 (0.23 – 0.41) | **<.001** | 0.48 (0.37 – 0.59) | | **<.001** | 0.31 (0.22 – 0.41) | **<.001** | 0.31 (0.21 – 0.40) | **<.001** | 0.46 (0.35 – 0.57) | **<.001** |
| Win Rate |  |  |  | |  | -0.11 (-0.39 – 0.17) | .428 |  |  |  |  |
| Domain [Loss] |  |  |  | |  |  |  | 8.99 (3.78 – 14.20) | **.001** | 7.69 (2.81 – 12.57) | **.002** |
| **Random Effects** | | | |  | | | | | | |  |
| σ^2^ | 182.75 | | 175.94 | | | 182.16 |  | 181.75 | | 173.85 | |
| τ_00_ | 296.46 _ppt_ | | 218.26 _ppt_ | | | 299.90 _ppt_ |  | 282.87 _ppt_ | | 210.30 _ppt_ | |
|  | 8.46 _timepoint_ | | 5.74 _timepoint_ | | | 7.87 _timepoint_ |  | 7.49 _timepoint_ | | 4.78 _timepoint_ | |
| ICC | 0.63 | | 0.56 | | | 0.63 |  | 0.62 | | 0.55 | |
| N | 201 _ppt_ | | 201 _ppt_ | | | 201 _ppt_ |  | 201 _ppt_ | | 201 _ppt_ | |
|  | 3 _timepoint_ | | 2 _timepoint_ | | | 3 _timepoint_ |  | 3 _timepoint_ | | 2 _timepoint_ | |
| Observations | 603 | | 402 | | | 603 |  | 603 | | 402 | |
| Marginal R^2^ / Conditional R^2^ | 0.195 / 0.698 | | 0.337 / 0.708 | | | 0.200 / 0.702 |  | 0.238 / 0.707 | | 0.365 / 0.716 | |

**Supplementary File 3B.** Excluding the final timepoint to investigate the effects of control, difficulty and stress during the WS Task for Study 1 (left hand model). Including all timepoints (as original model) and additionally including overall win rate as a covariate for Study 1 (right hand model).

|  | **Subjective Stress**  **(Study 1, excl. final timepoint)** | | **Subjective Stress**  **(Study 1, including win rate)** | |
| --- | --- | --- | --- | --- |
| *Predictors* | *Estimates*  *(95% CI)* | *p* | *Estimates*  *(95% CI)* | *p* |
| (Intercept) | 24.08 (17.39 – 30.76) | **<.001** | 34.92 (26.47 – 43.37) | **<.001** |
| Subjective Control | -0.09 (-0.16 – -0.02) | **.013** | -0.12 (-0.19 – -0.06) | **<.001** |
| Perceived Difficulty | 0.46 (0.39 – 0.54) | **<.001** | 0.37 (0.30 – 0.43) | **<.001** |
| Win Rate |  |  | -0.06 (-0.16 – 0.04) | .220 |
| σ^2^ | 189.22 | | 207.91 |  |
| τ_00_ | 340.94 _ppt_ | | 350.04 _ppt_ |  |
|  | 3.75 _timepoint_ | | 3.53 _timepoint_ |  |
| ICC | 0.65 | | 0.63 |  |
| N | 473 _ppt_ | | 473 _ppt_ |  |
|  | 3 _timepoint_ | | 4 _timepoint_ |  |
| Observations | 1419 | | 1892 |  |
| Marginal R^2^ / Conditional R^2^ | 0.184 / 0.711 | | 0.163 / 0.690 |  |
