# Supplementary material for: Sense of control buffers against stress
Source: eLife. 2026 Feb 10;14:RP105025. doi: 10.7554/eLife.105025 (PMC12890248; doi:10.7554/eLife.105025)
Supplement: Supplementary file 4. — (A) Linear mixed effects model for the stress relief when including initial stress level (after the WS/video task) as a covariate, predicting subjective stress from two timepoints: after the stressor and after the stressor debrief (timepoints 2 and 3). (B) Linear mixed effects models including total experiment time as a covariate, predicting subjective stress from two timepoints: before and after the stressor (stress induction, timepoints 1 and 2), and after the stressor and after the stressor debrief (stress relief, timepoints 2 and 3). (C) Linear mixed effects models including the interactions with Domain (rather than just as a covariate in the main analyses), predicting subjective stress from two timepoints: before and after the stressor (stress induction, timepoints 1 and 2), and after the stressor and after the stressor debrief (stress relief, timepoints 2 and 3). (D) Linear mixed effects models predicting subjective stress from two timepoints: before and after the stressor (stress induction, timepoints 1 and 2), and after the stressor and after the stressor debrief (stress relief, timepoints 2 and 3) in just the high control (WS task) condition, to test for the interactions with domain. [file elife-105025-supp4.docx]

**Supplementary File 4A.** Linear mixed effects model for the stress relief when including initial stress level (after the WS/video task) as a co-variate, predicting subjective stress from two timepoints: after the stressor and after the stressor debrief (timepoints 2 and 3).

|  | **Stress Relief** | |
| --- | --- | --- |
| *Predictors* | *Estimates (95% CI)* | *p* |
| (Intercept) | 93.53 (81.97 – 105.10) | **<.001** |
| Timepoint | -29.91 (-34.07 – -25.75) | **<.001** |
| Control [Neutral] | -10.65 (-30.21 – 8.90) | .285 |
| Stressor Intensity [Low] | -55.05 (-70.50 – -39.60) | **<.001** |
| Domain [Loss] | 0.24 (-4.88 – 5.35) | .928 |
| Initial Stress (after WS/video task) | 0.64 (0.57 – 0.72) | **<.001** |
| Timepoint × Control [Neutral] | 9.65 (2.32 – 16.99) | **.010** |
| Timepoint × Stressor Intensity [Low] | 19.87 (14.03 – 25.71) | **<.001** |
| Control [Neutral] × Stressor Intensity [Low] | 10.00 (-17.37 – 37.38) | .473 |
| (Timepoint × Control [Neutral]) × Stressor Intensity [Low] | -7.19 (-17.54 – 3.16) | .173 |
| **Random Effects** | | |
| σ^2^ | 222.45 | |
| τ_00_ _ppt_ | 216.85 | |
| ICC | 0.49 | |
| N _ppt_ | 295 | |
| Observations | 590 | |
| Marginal R^2^ / Conditional R^2^ | 0.495 / 0.745 | |

**Supplementary File 4B.** Linear mixed effects models including total experiment time as a co-variate, predicting subjective stress from two timepoints: before and after the stressor (stress induction, timepoints 1 and 2), and after the stressor and after the stressor debrief (stress relief, timepoints 2 and 3).

|  | **Stress Induction** | | **Stress Relief** | |
| --- | --- | --- | --- | --- |
| *Predictors* | *Estimates (95% CI)* | *p* | *Estimates (95% CI)* | *p* |
| (Intercept) | 20.68 (7.79 – 33.57) | **.002** | 118.64 (103.73 – 133.56) | **<.001** |
| Timepoint | 20.14 (15.30 – 24.99) | **<.001** | -29.91 (-34.07 – -25.75) | **<.001** |
| Control [Neutral] | -9.49 (-25.64 – 6.67) | .249 | -9.40 (-30.25 – 11.44) | .376 |
| Stressor Intensity [Low] | 12.87 (0.56 – 25.17) | **.040** | -56.20 (-72.36 – -40.04) | **<.001** |
| Domain [Loss] | 10.88 (3.99 – 17.77) | **.002** | 8.33 (1.42 – 15.24) | **.018** |
| Experiment Total Time | -0.00 (-0.00 – 0.00) | .087 | -0.00 (-0.00 – 0.00) | .335 |
| Timepoint × Control [Neutral] | 10.92 (2.38 – 19.46) | **.012** | 9.65 (2.32 – 16.99) | **.010** |
| Timepoint × Stressor Intensity [Low] | -14.66 (-21.46 – -7.86) | **<.001** | 19.87 (14.03 – 25.71) | **<.001** |
| Control [Neutral] × Stressor Intensity [Low] | 11.82 (-9.98 – 33.62) | .287 | 13.55 (-15.09 – 42.18) | .353 |
| (Timepoint × Control [Neutral]) × Stressor Intensity [Low] | -6.28 (-18.32 – 5.77) | .307 | -7.19 (-17.54 – 3.16) | .173 |
| **Random Effects** | | | | |
| σ^2^ | 301.09 | | 222.45 | |
| τ_00_ | 466.27 _ppt_ | | 510.42 _ppt_ | |
| ICC | 0.61 | | 0.70 | |
| N | 295 _ppt_ | | 295 _ppt_ | |
| Observations | 590 | | 590 | |
| Marginal R^2^ / Conditional R^2^ | 0.138 / 0.662 | | 0.164 / 0.746 | |

**Supplementary File 4C.** Linear mixed effects models including the interactions with Domain (rather than just as a covariate in the main analyses), predicting subjective stress from two timepoints: before and after the stressor (stress induction, timepoints 1 and 2), and after the stressor and after the stressor debrief (stress relief, timepoints 2 and 3).

|  | **Stress Induction** | | **Stress Relief** | |
| --- | --- | --- | --- | --- |
| *Predictors* | *Estimates (95% CI)* | *p* | *Estimates (95% CI)* | *p* |
| (Intercept) | 11.32 (-1.04 – 23.68) | .073 | 107.00 (90.80 – 123.20) | **<.001** |
| Timepoint | 20.86 (14.04 – 27.68) | **<.001** | -26.98 (-32.83 – -21.13) | **<.001** |
| Control [Neutral] | -10.68 (-28.44 – 7.08) | .238 | -3.72 (-26.99 – 19.54) | .753 |
| Stressor Intensity [Low] | 11.14 (-6.17 – 28.46) | .207 | -45.31 (-67.99 – -22.62) | **<.001** |
| Domain [Loss] | 14.01 (-3.57 – 31.58) | .118 | 22.94 (-0.08 – 45.96) | .051 |
| Timepoint × Control [Neutral] | 10.20 (0.40 – 20.01) | **.041** | 6.72 (-1.69 – 15.13) | .117 |
| Timepoint × Stressor Intensity [Low] | -12.73 (-22.28 – -3.17) | **.009** | 15.50 (7.30 – 23.70) | **<.001** |
| Control [Neutral] × Stressor Intensity [Low] | 13.31 (-11.69 – 38.30) | .296 | 2.52 (-30.23 – 35.27) | .880 |
| Timepoint × Domain [Loss] | -1.45 (-11.15 – 8.25) | .769 | -5.92 (-14.24 – 2.40) | .163 |
| Stressor Intensity [Low] × Domain [Loss] | 3.59 (-21.08 – 28.26) | .775 | -22.05 (-54.37 – 10.27) | .181 |
| (Timepoint × Control [Neutral]) × Stressor Intensity [Low] | -8.21 (-22.01 – 5.59) | .243 | -2.82 (-14.66 – 9.02) | .640 |
| (Timepoint × Stressor Intensity [Low]) × Domain [Loss] | -3.96 (-17.58 – 9.65) | .568 | 8.86 (-2.82 – 20.54) | .137 |
| **Random Effects** | | | | |
| σ^2^ | 301.79 | | 222.11 | |
| τ_00_ | 471.96 _ppt_ | | 512.60 _ppt_ | |
| ICC | 0.61 | | 0.70 | |
| N | 295 _ppt_ | | 295 _ppt_ | |
| Observations | 590 | | 590 | |
| Marginal R^2^ / Conditional R^2^ | 0.132 / 0.661 | | 0.162 / 0.747 | |

**Supplementary File 4D.** Linear mixed effects models predicting subjective stress from two timepoints: before and after the stressor (stress induction, timepoints 1 and 2), and after the stressor and after the stressor debrief (stress relief, timepoints 2 and 3) in just the high control (WS task) condition, to test for the interactions with domain.

|  | **Stress Induction** | | | **Stress Relief** | | |
| --- | --- | --- | --- | --- | --- | --- |
| *Predictors* | *Estimates (95% CI)* |  | *p* | *Estimates (95% CI)* |  | *p* |
| (Intercept) | 11.32 (-0.71 – 23.35) |  | .065 | 107.00 (90.43 – 123.57) |  | **<.001** |
| Timepoint | 20.86 (14.22 – 27.50) |  | **<.001** | -26.98 (-33.10 – -20.86) |  | **<.001** |
| Domain [Loss] | 14.01 (-3.09 – 31.10) |  | .108 | 22.94 (-0.62 – 46.49) |  | .056 |
| Stressor Intensity [Low] | 11.14 (-5.70 – 27.98) |  | .194 | -45.31 (-68.52 – -22.10) |  | **<.001** |
| Timepoint × Domain [Loss] | -1.45 (-10.89 – 7.99) |  | .763 | -5.92 (-14.61 – 2.78) |  | .182 |
| Timepoint × Stressor Intensity [Low] | -12.73 (-22.03 – -3.42) |  | **.007** | 15.50 (6.93 – 24.06) |  | **<.001** |
| Domain [Loss] × Stressor Intensity [Low] | 3.59 (-20.41 – 27.59) |  | .769 | -22.05 (-55.12 – 11.02) |  | .191 |
| (Timepoint × Domain [Loss]) × Stressor Intensity [Low] | -3.96 (-17.22 – 9.29) |  | .557 | 8.86 (-3.35 – 21.06) |  | .154 |
| **Random Effects** | | | | | | |
| σ^2^ | 285.34 | | | 241.92 | | |
| τ_00_ | 444.18 _ppt_ | | | 407.53 _ppt_ | | |
| ICC | 0.61 | | | 0.63 | | |
| N | 201 _ppt_ | | | 201 _ppt_ | | |
| Observations | 402 | | | 402 | | |
| Marginal R^2^ / Conditional R^2^ | 0.125 / 0.658 | | | 0.190 / 0.698 | | |
