# Supplementary material for: Sense of control buffers against stress
Source: eLife. 2026 Feb 10;14:RP105025. doi: 10.7554/eLife.105025 (PMC12890248; doi:10.7554/eLife.105025)
Supplement: Supplementary file 5. — (A) Descriptive statistics across the 4 conditions from Study 1. (B) Descriptive statistics across the 6 conditions in Study 2. Given that the Study 2 analyses compared group differences, we assessed group differences in demographic and questionnaire measures using a one-way ANOVA for continuous variables or a Chi-squared test for categorical variables. (C) Additional information about excluded participants. (D) Methodological details for both studies. [file elife-105025-supp5.docx]

**Supplementary File 5A.** Descriptive statistics across the 4 conditions from Study 1.

| ***Wheel Stopping Task Control*** | High | | Low | |
| --- | --- | --- | --- | --- |
| ***Stressor Intensity*** | High | Low | High | Low |
| **N** | 142 | 94 | 140 | 97 |
| **Age** – mean (SD) | 29.9 (7.70)^†^ | 32.1 (9.06) | 29.0 (5.94)^†^ | 30.3 (10.3)^†^ |
| **Female** – N (%) | 75 (0.53) | 40 (0.42)^†^ | 73 (0.52) | 53 (0.55) |
| **Nationality UK** – N (%) | 117 (0.84)^†^ | 76 (0.82)^†^ | 110 (0.80)^†^ | 74 (0.77) |
| **First language English** – N (%) | 123 (0.88)^†^ | 82 (0.87) | 114 (0.83)^†^ | 80 (0.84)^†^ |
| **LOC** – mean (SD) | 14.1 (3.94) | 12.7 (3.62) | 13.4 (3.60) | 13.9 (4.23) |
| **PHQ** – mean (SD) | 8.20 (5.71) | 5.24 (3.78) | 6.86 (5.16) | 5.4 (4.02) |
| **STAI State** (initial) – mean (SD) | 39.0 (11.3) | 34.5 (8.97) | 38.4 (10.9) | 36.8 (10.4) |
| **STAI Trait** – mean (SD) | 47.0 (11.8) | 43.1 (11.3) | 46.1 (11.8) | 46.1 (10.8) |
| **SPIN** – mean (SD) | 24.6 (14.6) | 20.9 (13.5) | 24.4 (15.3) | 23.6 (15.4) |

*Notes:* LOC = Locus of Control, PHQ = Patient Health Questionnaire, STAI = State-Trait Anxiety Inventory, SPIN = Social Phobia Inventory.
^†^Contains missing demographic data for some participants (max 3 per condition).

**Supplementary File 5B.** Descriptive statistics across the 6 conditions in Study 2. Given that the Study 2 analyses compared group differences, we assessed group differences in demographic and questionnaire measures using a one-way ANOVA for continuous variables or a Chi-squared test for categorical variables.

| Condition | *WS Task Control*  *Stressor Intensity*  *Domain* | High  High  Win | High  High  Loss | High  Low  Win | High  Low  Loss | Neutral  High  N/A | Neutral  Low  N/A | Group differences |
| --- | --- | --- | --- | --- | --- | --- | --- | --- |
| N | | 50 | 49 | 52 | 50 | 47 | 47 |  |
| Ethnicity – N (%) Asian  Black  Mixed  Other  White | | 2 (4.0)  6 (12.0)  4 (8.0)  2 (4.0)  36 (72.0) | 8 (16.3)  2 (4.1)  2 (4.1)  0 (0)  37 (75.5) | 8 (16.0)  4 (8.0)  1 (2.0)  1 (2.0)  36 (72.0) ^†^ | 4 (8.2)  5 (10.2)  2 (4.1)  0 (0)  38 (77.6) ^†^ | 5 (10.6)  4 (8.5)  2 (4.3)  2 (4.3)  34 (72.3) | 3 (6.5)  3 (6.5)  1 (2.2)  1 (2.2)  38 (82.6) ^†^ | X(20, N=291) =15.76, *p*=0.732 |
| Age – mean (SD) | | 29.1 (5.66) | 27.4 (4.50) | 28.5 (4.58)^†^ | 29.2 (4.70)^†^ | 28.6 (4.92) | 28.9 (4.59) | *F*(5, 287) = 0.84, *p*=.522 |
| Female – N (%) | | 25 (0.5) | 25 (0.51) | 26 (0.51)^†^ | 24 (0.49)^†^ | 25 (0.53) | 23 (0.49) | X(5, N=293) = 0.24, *p*=.999 |
| Nationality UK – N (%) | | 38 (0.76) | 44 (0.90) | 42 (0.84)^†^ | 41 (0.84)^†^ | 37 (0.79) | 37 (0.79) | X(5, N=292) = 4.12, *p*=.533 |
| First language English – N (%) | | 42 (0.84) | 45 (0.92) | 42 (0.82)^†^ | 43 (0.88)^†^ | 39 (0.83) | 44 (0.94) | X(5, N=293) = 4.91, *p*=.427 |
| LOC – mean (SD) | | 14.4 (3.83) | 14.6 (3.66) | 14.1 (3.69) | 14.5 (4.06) | 14.1 (4.10) | 13.6 (3.90) | *F*(5, 286) = 0.41, *p*=.841 |
| PHQ – mean (SD) | | 6.98 (6.3) | 8.31 (5.33) | 5.42 (4.34) | 7.42 (5.92) | 6.87 (5.02) | 7.47 (6.22) | *F*(5, 289) = 1.50, *p*=.189 |
| STAI State (initial) – mean (SD) | | 37.9 (12.6) | 42.0 (12.3) | 37.0 (10.2) | 38.3 (11.8) | 38.8 (10.9) | 40.0 (14.4) | *F*(5, 287) = 1.07, *p*=.377 |
| STAI Trait – mean (SD) | | 45 (14.0) | 48.4 (11.6) | 43.6 (10.6) | 45.6 (11.7) | 46.0 (9.99) | 47.8 (14.6) | *F*(5, 284) = 1.05, *p*=.390 |
| SPIN – mean (SD) | | 20.6 (14.2) | 25.1 (13.1) | 18.7 (11.7) | 23.0 (14.1) | 23.7 (12.4) | 22.9 (14.4) | *F*(5, 284) = 1.49, *p*=.192 |

*Notes:* LOC = Locus of Control, PHQ = Patient Health Questionnaire, STAI = State-Trait Anxiety Inventory, SPIN = Social Phobia Inventory. ^†^Contains missing demographic data for 1-2 participants.

**Supplementary File 5C.** Additional information about excluded participants.

Here we provide additional information about the twelve participants who were excluded from the final sample. Four participants had duplicate data and were excluded from analyses. Of these, two of these participants completed the same experimental version twice (both therefore excluded), despite using Prolific settings to aim to exclude participants who had already completed previous iterations of the study. The other two participants had each completed two different experimental conditions (as the conditions were released sequentially), and so data from their second participation was excluded. Overall, four participants failed more than half of the questionnaire attention checks, but only two of these were excluded entirely. Two of these had a win rate (of the main WS task) below 1SD of the mean for that version and were excluded due to questionable data quality. The other two had reasonable task performance (win rate within 1SD below the mean; or passed video attention checks in the ‘neutral’ control condition) so were not excluded (questionnaires with failed attention checks still receive a missing value for that questionnaire). Six participants were excluded from Study 2 video condition as they failed less than 3/6 video attention checks.

**Supplementary File 5D.** Methodological details for both studies.

|  | **Study 1** | **Study 2** |
| --- | --- | --- |
| Main Paradigm Summarised | Stressor then WS task | Mild stressor, WS task or Video task, then stressor |
| Conditions | 4: WS Task Control (High v Low) + Stressor Intensity (High v Low) | 6: Control (WS High v Neutral) + Stressor Intensity (High v Low), for High Control, also Domain condition (Win v Loss) |
| Procedure (order of tasks) | 1. PHQ 2. SPIN 3. LOC 4. STAI-S 5. STAI-T 6. Stress slider 7. Stressor 8. STAI-S 9. Stress slider 10. Stressor debrief 11. STAI-S 12. Stress slider 13. WS task 14. STAI-S 15. Stress slider 16. Final debrief | 1. PHQ 2. SPIN 3. LOC 4. STAI-S 5. STAI-T 6. Stress slider 7. Mild stress instructions 8. STAI-S 9. Stress slider 10. WS task / Video task 11. STAI-S 12. Stress slider 13. Stressor 14. STAI-S 15. Stress slider 16. Stressor debrief 17. STAI-S 18. Stress slider 19. Final debrief |
| N WS Trials Total | 320 | 135 |
| N Blocks & WS Task Parameter Presentation | 64, randomised order | 27, 3 sets of 3 blocks in increasing control (randomised order within set) |
| Control Slider Frequency | After the first block (5 trials), then every 2 blocks (10 trials) thereafter. Total of 32 sliders. | After the first block (5 trials), then every 2 blocks (10 trials) thereafter. Total of 14 sliders  (n/a for videos) |
| Difficulty Slider Frequency | After the second block (10 trials), then every 2 blocks (10 trials) thereafter, alternating with control slider. Total of 32 sliders. | After the second block (10 trials), then every 2 blocks (10 trials) thereafter, alternating with control slider. Total of 13 sliders  (n/a for videos) |
| Stress Slider Frequency | Within WS task after the 17^th^ block, then every 16 blocks thereafter (except after final block), plus 4 times outside of WS task. Total of 7 sliders | Within WS task after the 9^th^ block, then every 9 blocks thereafter (except after final block), plus 5 times outside of WS task.  For videos, after each block plus 5 times outside of video section. Total of 7 sliders. |
| £ Domain | Win (1p per correct trial) | WS task: Win (1p per correct trial) (n=102) or lose £3 bonus if incorrect trial selected (n=99).  Videos: Win (participants told bonus for all attention questions correct, all received £1 regardless) |
| Trial-by-trial feedback | Correct: “Congratulations! You have won an additional 0.01 GBP for this trial.”  Incorrect: “You have lost. You will receive an additional 0.00 GBP for this trial.” | Win domain:  Correct: “Congratulations! You have won an additional 0.01 GBP for this trial.”  Incorrect: “You have lost. You will receive an additional 0.00 GBP for this trial.”  Loss domain:  Correct: “Success!”  Incorrect: “Fail!”  NB: Loss domain practice trial feedback:  Correct: “Success! If this trial was selected as the bonus trial, you would have kept all your bonus money!”  Incorrect: “"Fail. If this trial was selected as the bonus trial, you would have lost all your bonus money.” |
| Practice trials | No | No (Win domain)  Yes (Loss domain, 6 trials) |
| Task Parameters |  |  |
| Speeds (High Control) | 0.06, 0.08, 0.1, 0.12 | 0.06, 0.09, 0.12 |
| Speeds (Low Control) | 0.15, 0.2, 0.25, 0.3 | n/a |
| Segment Size Fractions (High Control) | 0.6, 0.7, 0.8, 0.9 | 0.6, 0.7, 0.8, 0.9 |
| Segment Size Fractions (Low Control) | 0.6, 0.7, 0.8, 0.9 | n/a |
| Stopping Angles (High Control) | 0.1π, 0.2π, 0.4π, 0.8π | 0.1π, 0.3π, 0.8π |
| Deceleration Increments (Low Control) | 0.0002, 0.0004, 0.0006, 0.0008 | n/a |

*Notes:* For a subset of Study 1 (37%) participants were not presented with the PHQ, SPIN or LOC questionnaires at the start of the experiment, the control sliders were asked every 4 blocks (20 trials) (total of 16 sliders) and difficulty sliders were presented every block except every 4th (i.e. when not control slider) (5-10 trials) (total of 48 sliders). Additionally, in the remaining sample of Study 1, half of the participants in the High Stressor Intensity condition (n=99) received items 10, 11, 12 (the stress debrief) just before the final debrief, as a ‘late debrief’ condition. All participants are included irrespective of the timing of the debrief in the analyses given that we do not investigate the effects of the stressor from Study 1.
